# 组织驻留记忆T细胞在肺癌中的研究进展

**DOI:** 10.3779/j.issn.1009-3419.2022.102.49

**Published:** 2022-12-20

**Authors:** 思含 谈, 攀文 田

**Affiliations:** 610041 成都，四川大学华西医院呼吸与危重症医学科/肺癌中心 Department of Respiratory and Critical Care Medicine/Lung Cancer Center, West China Hospital, Sichuan University, Chengdu 610041, China

**Keywords:** 组织驻留记忆T细胞, 肺肿瘤, 免疫治疗, Tissue-resident memory T cell, Lung neoplasms, Immunotherapy

## Abstract

免疫检查点抑制剂（immune checkpoint inhibitors, ICIs）虽然广泛用于肺癌的治疗，但获益人群有限，且缺乏有效的疗效预测标志物。组织驻留记忆T细胞（tissue-resident memory T cell, TRM）通过表达整合素CD103、CD49a或C型凝集素CD69和免疫检查点受体，使其在组织中驻留，并发挥抗肿瘤作用。表达程序性死亡受体1（programmed cell death 1, PD-1）的TRM富含与细胞毒性相关的转录产物，增强了T细胞（抗原）受体（T cell receptor, TCR）介导的细胞毒作用。TRM可以预测肺癌患者免疫治疗疗效和预后，是具有前景的生物标志物。本综述将阐述TRM在肺癌中的研究进展。

## Abstract (en)

Immune checkpoint inhibitors (ICIs) have been widely used in the treatment of lung cancer, but the benefit population is limited and there is a lack of effective predictive markers of efficacy. Tissue-resident memory T cells (TRM) reside in tissues and exert anti-tumor effects by expressing the integrins CD103, CD49a or C-type lectin CD69 and immune checkpoint receptors. TRM expressing programmed cell death 1 (PD-1) is enriched with transcriptional products associated with cytotoxicity and enhances T cell (antigen) receptor (TCR)-mediated cytotoxicity. TRM is a promising biomarker for predicting the efficacy and prognosis of immunotherapy in lung cancer patients. This review will describe the progress of TRM research in lung cancer.

肺癌仍是当前全球癌症相关死亡的首要原因。尽管应用免疫检查点抑制剂（immune checkpoint inhibitors, ICIs）极大地改善了肺癌患者的预后，但能从ICIs治疗中长期获益的非小细胞肺癌（non-small cell lung cancer, NSCLC）患者不足20%^[1]^。目前肺癌患者接受ICIs治疗缺乏有效的预测疗效和评估预后的生物标志物。

组织驻留记忆T细胞（tissue-resident memory T cell, TRM）属于特别的淋巴细胞谱系，驻留于组织中而不进入血液再循环^[2]^。TRM在参与抗肿瘤治疗中发挥了重要作用，可作为肺癌患者的免疫治疗疗效预测和预后标志物。本文将围绕TRM的定义、分类分型、来源与分化、组织驻留性、调控机制、在肺癌免疫治疗中疗效预测价值和预后价值进行阐述。

## 1 TRM的概述

### 1.1 TRM的定义与分类

TRM是一种具有整合素CD103、CD49a或C型凝集素CD69的特定组织驻留表型^[3]^，而缺乏组织外迁标志物如鞘氨醇-1-磷酸受体（sphingosine-1-phosphate receptor 1, S1PR1）、CD62L和CCR7的记忆性T细胞亚群^[4-6]^。TRM存在于各种肿瘤组织中，如NSCLC、卵巢癌和乳腺癌^[3]^，是一类非循环记忆T细胞^[4]^，包括CD8^+^ TRM、CD4^+^ TRM、Treg TRM、γδT TRM和自然杀伤（natural killer, NK）TRM等。TRM在抗感染、抗肿瘤的治疗以及自身免疫性疾病的致病机制中发挥重要作用^[7, 8]^。TRM的分类、细胞表面分子、存在部位及对应功能详见表 1^[7, 9-11]^。

**表 1:** TRM的分类 The classification of TRM

Cellular classification	Cell surface molecules	Position	Function
CD8^+^ TRM^[9]^	CD69, CD103	Epithelial layers of barrier tissues	(1) Functioning before circulating memory CD8^+^ T cells; (2) Act as sentinels to trigger antigen-specific protection against reinfection
CD4^+^ TRM^[9]^	CD69	Below the epithelial layers，cluster in lymphoid structures	Optimize interactions with antigen-presenting cells upon reinfection
Treg TRM^[7]^	CD69, CD103	Autoimmune diseases and chronic infected inflammatory sites	Regulates immune response and maintains body homeostasis
*γδ*T TRM^[10]^	CD69, CD103	Skin, gut, lungs and reproductive tract	Recognizes non-peptide antigens and stress-induced production of ligands that play an important role in mucosal immunity
NK TRM^[11]^	CD103, CD49a	Liver, lung, bone marrow and adipose tissue	(1) Recognize glycolipid antigen in the context of CD1d; (2) Play a role in tumor surveillance and the control of some viral and bacterial infections
TRM: tissue-resident memory T cell; NK: natural killer.			

### 1.2 TRM来源与分化

记忆T淋巴细胞根据经典分类可分为两类，即中央记忆T细胞（central memory T cell, TCM）和效应记忆T细胞（effector memory T cell, TEM）。近年来发现了记忆T淋巴细胞的第三个亚群被称为TRM，它不经血液再循环而存在于组织中，是屏障部位（如皮肤、肺、肠道黏膜）的“第一道防线”^[12]^。

部分淋巴组织的TRM可能由TCM分化而来，外周组织的TRM可能来源于经转化生长因子β（transforming growth factor β, TGF-β）及肿瘤抗原作用而分化的TEM或效应T细胞^[4]^，并且外周组织中的TRM因其不具有参与血液循环或转化为其他记忆性T细胞亚群的特性，属于终末分化的细胞^[13]^。TRM的形成、扩增和长期维持均需要抗原的刺激^[14]^。

### 1.3 TRM的组织驻留性

TRM表达广泛的整合素^[5]^，如CD103、CD69、CD44和CD49a，以及趋化因子受体如CXCR3和CXCR6^[15]^，同时缺乏淋巴结归巢受体CD62L和CCR7^[5]^，这些表面分子促进TRM向肿瘤部位迁移，维持TRM在肿瘤组织中的驻留性。

CD103作为TRM的关键表面标志物，主要在CD8^+^ TRM和部分CD4^+^ TRM上表达，与E-钙黏蛋白牢固黏附，触发富含脯氨酸的酪氨酸激酶2和桩蛋白衔接蛋白的磷酸化，启动整合素由外向内的信号传导^[16]^，有助于T细胞在上皮内的黏膜特异性滞留^[5, 17-19]^，也有助于CCR5在T细胞和肿瘤细胞之间形成的免疫突触处募集，减弱T细胞受肿瘤组织以外的CCL5趋化作用，从而促进TRM在肿瘤组织的驻留^[20]^。TRM表达CD103等黏附受体，与周围肿瘤细胞结合，使TRM难以进入循环，从而驻留于肿瘤组织^[21]^。

CD69与TRM表面参与组织外迁的受体S1PR1结合，导致S1PR1的内化和降解，抑制干扰素α（interferon α, IFN-α）/干扰素β（interferon β, IFN-β）下游S1PR的表达^[22]^，使TRM丧失对血液和淋巴管中的鞘氨醇-1-磷酸（sphingosine-1-phosphate, S1P）反应能力，阻止S1P介导的T细胞外流^[23, 24]^，从而阻碍T细胞从肿瘤组织迁移到血液和淋巴组织中^[6, 25]^。CD69缺陷型CD8^+^ T细胞在皮肤和肺中都显示出TRM发育缺陷^[23, 26]^。研究^[27, 28]^表明，CD69不宜作为TRM的可靠标志物，特别在外周组织，因部分CD69^+^ T细胞在TRM组织外迁信号S1PR1、Krüppel样转录因子2（Krüppel-like transcription factor 2, KLF2）、T细胞因子1（T cell factor 1, TCF1）等沉默之前，仍可进入循环。

CD44是TRM的另一个表面标志物。非激酶跨膜糖蛋白CD44在肿瘤干细胞等多种细胞类型中过度表达^[29]^。CD44作为透明质酸等细胞外基质的受体^[30]^，可与透明质酸结合，介导细胞与细胞外基质之间的黏附，在诱导记忆T细胞在肿瘤组织中的驻留发挥作用^[31]^。

TRM也表达高水平的CD49a，CD49a构成了α1β1整合素的α亚基，与β1亚基和基底膜上的IV型胶原结合后^[32]^，使得TRM在组织中驻留，如在人的肺和血液中的CD49a^+^CD56^+^CD16^-^ NK细胞具有组织驻留的特征^[33]^。在黑色素瘤小鼠模型中，CD49a定义了皮肤中的细胞毒性CD8^+^ TRM^[32, 34]^。

在NSCLC中，与CD8^+^CD103^-^肿瘤浸润淋巴细胞（tumor infiltrating lymphocytes, TILs）相比，CD8^+^ TRM高度表达CD39^[35]^。在转移性淋巴结中也检测到CD39^+^CD8^+^ TRM，但在非肿瘤累及的淋巴结或循环血中并未检测到，表明CD39^+^CD8^+^ TRM是区分肿瘤的特异性标志^[36]^。

TRM在组织中的驻留性还可归因于存在招募TRM前体细胞到外周组织的趋化因子受体CXCR6^[19, 32, 37-39]^，缺乏引导细胞进入淋巴组织的淋巴归巢受体CCR7和CD62L^[40]^，缺乏介导内皮细胞转运的趋化因子受体CX3CR1，使TRM不再与内皮细胞相互作用后而发生迁移^[15]^。由此可见，TRM的组织驻留性是由具驻留特性的表面分子和缺乏归巢趋化受体共同作用的结果。

### 1.4 TRM的调控机制

TRM主要由TGF-β、肿瘤坏死因子α（tumor necrosis factor α, TNF-α）、IL-15、IL-2、IL-12和IL-33等细胞因子调控。

#### 1.4.1 TGF-β在TRM的形成中起关键作用

TGF-β诱导CD103、CD69的表达^[4, 27]^，促进TRM在组织中的形成^[3]^。TGF-β将转录因子Smad2/3及NFAT-1与编码CD103（αE）亚单位的*ITGAE*基因的启动子和增强子元件结合^[41]^，并与特定的肿瘤多肽-主要组织相容性复合体-I（major histocompatibility complex I, MHC-I）复合体结合，直接参与肿瘤特异性T细胞CD103的表达^[3]^。TGF-β激活整合素连接激酶和蛋白激酶途径，启动整合素由内向外的信号传导，使得CD103和E-钙黏蛋白相互作用加强^[42]^。此外，在富含TGF-β的肿瘤微环境中肿瘤特异性T细胞与同源抗原结合后，可诱导和促进整合素CD103的表达以形成TRM^[43]^，此后肿瘤抗原的频繁刺激能够维持CD8^+^ TRM上CD103的高表达水平^[19]^，因此TRM在肿瘤微环境中得以形成并维持。

#### 1.4.2 TGF-β通过多种途径维持TRM的存活和功能

TGF-β参与抑制TILs上整合素LFA-1的表达^[19, 41, 42]^；诱导上皮细胞中Notch配体的表达，传导维持TRM存活关键的Notch信号。Notch信号通过调节葡萄糖代谢、氨基酸、微量元素和离子转运蛋白等，维持TRM上CD103的表达^[15]^，发挥CD103抗感染、抗肿瘤的功能。TGF-β下调T-box转录因子Eomes和T-bet，Eomes的表达在TRM发育过程中逐渐消退，而低水平T-bet促进IL-15Rβ链（CD122）的表达^[27]^，对TRM的存活和功能至关重要^[2, 43]^。T-bet下调后，TGF-β信号通路中CD103表达上调^[44]^，促进TNF-α、干扰素γ（interferon γ, IFN-γ）和颗粒酶B等因子的表达，从而增强CD8^+^ TRM的杀伤作用^[44]^。由于Eomes和T-bet转录因子都是TGF-β受体表达的负性调控因子，T-bet和Eomes的下调使TGF-β反应性增加，从而形成一个反馈回路，促进TRM分化^[11, 27]^。以上分子的表达会导致其他转录因子沉默，如KLF2、TCF1和参与TRM组织外迁的受体（如S1PR1），抑制TRM从组织中迁移，维持TRM的驻留性^[45]^。

#### 1.4.3 TGF-β与其他因子的协同作用

TGF-β促进由T-bet和IL-15调控的Hobit表达^[46]^，通过IL-2和IL-12以T-bet非依赖性方式诱导Blimp 1的表达^[45, 47]^。Hobit或Blimp 1的缺乏导致定位组织的CD8^+^ T细胞减少，如果两者同时缺失可近乎完全阻断皮肤、肠道、肾和肝脏中TRM发育^[11]^。TGF-β、IL-33和TNF通过磷脂酰肌醇3-激酶-蛋白激酶B依赖的途径发挥作用，抑制KLF2的表达，进而下调S1PR1，增加CD69的表达^[3, 4, 24, 43]^，促进CD8^+^ TRM的形成^[11]^。

## 2 TRM在肿瘤微环境中的作用

TRM具有抑制肿瘤进展的作用^[6, 48, 49]^，在肿瘤的免疫监视中发挥着重要作用^[35, 50]^。CD8^+^ TRM亚群表达整合素CD103和免疫检查点受体，表现出更强的增殖、细胞因子分泌、细胞毒性和肿瘤杀伤等抗肿瘤效应^[19, 51-53]^。CD8^+^ TRM是肺癌肿瘤微环境中特异性激活并发挥长期抗肿瘤免疫反应的关键细胞亚群^[54]^。

### 2.1 整合素CD103在抑制肿瘤中发挥的作用

CD103与肿瘤细胞上的E-钙黏蛋白结合^[55]^，通过T细胞与特定肿瘤细胞的黏附来促进抗原识别^[56]^，在免疫突触处诱导细胞毒颗粒极化^[34, 57]^，提高T细胞（抗原）受体（T cell receptor, TCR）的敏感性^[58]^，增强TCR依赖的靶细胞杀伤^[3]^。CD8^+^CD103^+^ TRM增殖能力强，释放更多的细胞因子，如促炎细胞因子，吸引其他免疫细胞或细胞毒介质来消除肿瘤细胞^[21]^，表现出更强的肿瘤抑制能力^[3, 59]^。此外，Duhen等^[36]^提出CD8^+^CD39^+^CD103^+^ TRM具有独特的TCR库，在肿瘤中T细胞克隆扩增，还以MHC-I类依赖性方式有效杀死自体肿瘤细胞。

### 2.2 免疫检查点受体在抑制肺癌发展中的作用

与其他肿瘤浸润性T细胞相比，CD103^+^ TRM表达多种免疫检查点受体，如程序性死亡受体1（programmed cell death 1, PD-1）、淋巴细胞活化基因-3（lymphocyte activation gene-3, LAG-3）、T细胞免疫球蛋白黏蛋白分子-3（T cell immunoglobulin and mucin-containing molecule-3, TIM-3）和细胞毒性T淋巴细胞相关抗原-4（cytotoxic T lymphocyte-associated antigen-4, CTLA-4）^[19, 35, 52, 60-64]^，而NSCLC来源的TRM不表达CTLA-4^[19]^，与其维持自身抗原耐受的能力相关^[40, 65]^。

在NSCLC中，TRM表面的PD-1与封闭抗体的中和作用增强了CD103依赖的TCR介导的对自体肿瘤细胞的细胞毒作用^[3, 19]^。此外，肺癌中表达PD-1的TRM克隆扩增，并富含与细胞增殖和细胞毒性相关的转录产物，这一特征在共表达PD-1和TIM-3的TRM亚群中更为突出^[66]^。尽管CD103^+^ TRM表达PD-1，但并不是耗竭细胞^[52, 67]^，相反其抗肿瘤效能更强^[68-73]^。

除了TRM表面整合素CD103和免疫检查点受体在抑制肺癌中发挥了重要作用，TGF-β的诱导^[20, 34, 74]^、TRM细胞因子的分泌^[36, 60, 66]^及TRM直接和间接的细胞毒作用也参与了TRM的抗肿瘤机制^[19, 35, 75]^。

## 3 TRM与肺癌治疗

### 3.1 TRM与肺癌的过继细胞转移疗法（adoptive cell transfer therapy, ACT）

ACT的基本原理是提高抑制肿瘤细胞生长和存活细胞的数量和质量，破坏肿瘤细胞的免疫耐受性，其中淋巴细胞从患者体内提取，在体外进行修饰或扩增，然后转移到患者体内^[76]^，因其可以在体内激活和增殖，具有极其持久的抗肿瘤作用^[77]^。采用CD8^+^ TRM的ACT提高了免疫治疗疗效^[43]^。一项Ⅰ期临床研究^[78]^表明，对手术、放射治疗、化疗、综合治疗无效或不耐受的晚期或复发NSCLC患者重新输入体外唑来膦酸盐扩增的CD69^+^NKG2D^+^γδ TRM后其中位无进展生存期约4个月，相较对一种或两种化疗方案失败后使用吉非替尼/多西他赛中位无进展生存期（2个月）更长，在随后的研究中发现血浆IFN-γ升高，对肺癌细胞产生细胞毒作用。

ACT借助体外修饰扩增后的淋巴细胞输入免疫受损的肿瘤患者体内，改善患者原肿瘤微环境中免疫抑制状态，使淋巴细胞增殖，释放细胞因子，对肿瘤细胞的发生发展起到遏制作用。

### 3.2 TRM可作为肺癌免疫治疗疗效标志物

在接受抗PD-1/程序性死亡配体1（programmed cell death ligand 1, PD-L1）治疗的NSCLC患者中，对治疗有反应的患者其肿瘤内高表达ITGAE^[61]^，肿瘤微环境中CD8^+^CD103^+^ TRM亚群增加，但无反应患者的肿瘤微环境中CD8^+^CD103^+^ TRM亚群不增加^[59]^，表明CD8^+^CD103^+^ TRM与免疫治疗的良好反应密切相关^[61]^。

CD8^+^CD103^+^ TRM显著增高的肺癌患者接受抗PD-1/PD-L1治疗有较好的反应，可能归因于以下机制：①TRM表达整合素CD103诱导细胞毒颗粒极化，分泌颗粒酶B、穿孔素、IFN-γ、IL-17等因子，吸引其他免疫细胞或细胞毒介质来消除肿瘤细胞^[21]^；②CD8^+^CD103^+^ TRM表达免疫检查点受体，如PD-1、LAG-3、TIM-3^[19, 35]^，免疫细胞亦表达更高水平的PD-L1^[61]^，在接受PD-1/PD-L1阻断剂治疗时，增强CD103依赖的TCR介导的对自体肿瘤细胞的细胞毒作用^[3, 19]^，更显著地打破原PD-1信号诱导下的免疫抑制状态^[61]^；③在PD-1/PD-L1阻断剂作用下，CD8^+^CD103^+^ TRM克隆扩增^[61, 66]^并产生更多具有细胞毒作用的IFN-γ^[52]^，发生抗体依赖的细胞介导的细胞毒性作用（antibody dependent cell mediated cytotoxicity, ADCC）^[19]^，若将PD-L1阻断剂与肿瘤坏死因子受体超家族的4-1BB激动剂相结合能放大这一作用，更好地促进肿瘤消退^[79]^。

对肺癌组织中高度浸润CD8^+^CD103^+^ TRM的患者使用ICIs治疗，能获得良好临床反应，提示CD8^+^CD103^+^ TRM可作为抗PD-1/PD-L1治疗疗效的生物标志物^[3, 55]^，也是早期免疫反应的独特标志^[67]^。除了阻断PD-1/PD-L1，对TRM表面TIM-3的阻断可为抗PD-1耐药的肿瘤提供新的治疗方法^[55, 66]^。

## 4 TRM是肺癌患者良好预后的生物标志物

在两个早期肺癌患者的队列中，CD8^+^ TRM的高度浸润与患者无病生存期和总生存期（overall survival, OS）相关^[19, 35]^。在接受PD-L1抗体阿替利珠单抗治疗的肺癌队列中，与CD103^+^ TRM低浸润患者相比，CD103^+^ TRM高度浸润患者的OS显著延长5.5个月^[61]^。另一项结合TCGA数据集中372例接受抗PD-1/PD-L1治疗肺腺癌的研究证实TRM高度浸润的肺腺癌患者OS显著延长^[67]^。在一个以早期肺癌为主的队列^[35]^中，与高密度细胞毒性T淋巴细胞（cytotoxic T lymphocyte, CTL）相比，CD103^+^ TRM高度浸润的肺癌患者死亡率显著降低。即使在高密度CTL的肺癌患者中，CD103^+^ TRM高度浸润的患者死亡率显著降低，且存活时间显著延长。除CD8^+^ TRM外，CD4^+^ TRM在NSCLC中的浸润也是增加生存率的有利预测因子^[36]^。应用影像学发现CD8^+^ TRM高度浸润组患者淋巴结受累明显少于低度浸润组，间接反映了TRM的存在能够抑制肿瘤细胞淋巴结的转移和影响患者的临床分期^[54]^。

结合以上临床和影像数据证实TRM的存在与肺癌患者较好的预后相关，且较高TRM的数量和密度能更好地反映肺癌患者预后，这种影响与CTL无关^[35, 80]^。

整合素CD103可定义为肺癌患者良好预后的生物标志物^[35, 55, 67, 80]^。肿瘤组织中同时存在继发性滤泡样的三级淋巴结构（tertitary lymphoid structure, TLS）和高比例的CD103^+^ TRM更有利于评估肺腺癌患者的预后^[81]^。

由此可见，CD103^+^ TRM高度浸润与肺癌患者更好的生存结局相关，可作为肺癌患者的预后标志物，联合其他指标可提升预后评估效能。

## 5 总结与展望

TRM因其在肿瘤中高度浸润，表达整合素CD103诱导细胞毒颗粒极化，释放细胞因子，与ICIs结合后克隆扩增并发挥CD103依赖的TCR介导的对肿瘤细胞的细胞毒作用，改变抑制状态的肿瘤免疫微环境。在肺癌患者的治疗中，TRM能够预测免疫治疗疗效，TRM上的整合素CD103可作为肺癌患者良好预后的生物标志物。如何上调CD8^+^ T细胞表面整合素CD103的表达，增加肿瘤组织中TRM的数量和密度，发挥TRM表面免疫检查点受体的效力，提高免疫治疗的疗效，克服免疫治疗的耐药是抗肿瘤研究的重要方向。TRM在肺癌ACT中的价值、肺癌免疫治疗中的疗效预测价值、预后标志物的价值，均有待更多的研究来探索和证实。
